# C-terminal in Sp1-like artificial zinc-finger proteins plays crucial roles in determining their DNA binding affinity

**DOI:** 10.1186/1472-6750-13-106

**Published:** 2013-12-01

**Authors:** Baozhen Zhang, Shengyan Xiang, Yanru Yin, Liankun Gu, Dajun Deng

**Affiliations:** 1 Key laboratory of Carcinogenesis and Translational Research (Ministry of Education), Division of Etiology, Peking University Cancer Hospital & Institute, Beijing 100142, China

## Abstract

**Background:**

It is well known that the *C-*terminal zinc-finger-3 in transcription factor Sp1 contributes more than the *N-*terminal zinc-finger-1 in determining Sp1’s DNA binding capacity. Sp1-like artificial poly-zinc-finger proteins (ZFPs) are powerful biotechnological tools for gene-specific recognization and manipulation. It is important to understand whether the *C-*terminal fingers in the Sp1-like artificial ZFPs remain crucial for their DNA binding ability. Recently, a set of *p16* promoter-specific seven-ZFPs (7ZFPs) has been constructed to reactivate the expression of methylation-silenced *p16*. These 7ZFPs contain one *N-*terminal three-zinc-finger domain of Sp1 (3ZF), two Sp1-like two-zinc-finger domains derived from the Sp1 finger-2 and finger-3 (2ZF) in the middle and *C-*terminal regions.

**Results:**

In the present study, sets of variants for several representative 7ZFPs with the *p16-*binding affinity were further constructed. This was accomplished through finger replacements and key amino acid mutations in the *N*-terminal fingers, *C*-terminal fingers, and linker peptide, respectively. Their *p16-*binding activity was analysed using gel mobility shift assays. Results showed that the motif replacement or a key amino acid mutation (S > R) at position +2 of the α-helix in the *C-*terminal 2ZF domain completely abolished their *p16-*binding affinity. Deletion of three amino acids in a consensus linker (TGEKP > TG) between *finger-7* and the 6 × Histidine-tag in the *C-*terminal also dramatically abolished their binding affinity. In contrast, the replacement of the *finger-3* in the *N*-terminal 3ZF domain did not affect their binding affinity, but decreased their binding stability.

**Conclusions:**

Altogether, the present study show that the *C-*terminal region may play crucial roles in determining the DNA binding affinity of Sp1-like artificial ZFPs.

## Background

The C2H2 zinc-finger is one of the most common DNA-binding motifs in eukaryotes. Each C2H2 zinc-finger domain is a simple ββα-fold peptide and consists of about 30 amino acid residues in length. Structure stability of this fold is achieved by hydrophobic interaction by chelating of a single zinc ion with the conserved cysteine and histidine residues [1]. Nucleic acid recognition of zinc-finger proteins (ZFPs) is achieved through key amino acid residues at positions −1, +2, +3, and +6 of the α-helix in each finger, which typically bind with three adjacent bases (a triplet) [2,3]. A simple mode of DNA recognition by the C2H2 zinc-finger domain provides an ideal scaffold for designing proteins with novel sequence specificities. To achieve high binding specificity in the context of a large genome, longer arrays of zinc-fingers are required [4]. The ability of artificial ZFPs to target specific DNA sequences has led to the development of chimerical DNA modifying enzymes and transcription factors for specific genes [5-9]. This in turn has opened the possibility of using the engineered ZFP-based factors as novel human therapeutics. Compared with two recently established CRISPR and TALE technologies [10,11], the Sp1-like ZFP transcription factors may still be the best choice for gene transcription therapy because of their low antigenicity for human beings.

It has been reported that *C-*terminal *finger-2* and *finger-3* in transcription factor Sp1 contribute more than *N-*terminal *finger-1* when targeting GC-box DNA [12]. Similar phenomenon has also been observed in Kaiso and other C2H2 zinc-finger protein [13,14]. However, it is not well studied whether the *C-*terminal domain in artificial ZFPs is still a crucial determinant for their DNA binding affinity. In addition, amino acid at position +2 of the α-helix in zinc fingers might interact with the 4^th^ DNA base on the opposite strand of the flanking triplet DNA target. This amino acid/base contact enables synergy between adjacent fingers in sequence-specific DNA recognition for natural ZFPs such as tramtrack, Zif268, and Sp1 [15-17]. It is not clear whether the amino acid at position +2 in artificial ZFPs plays a role in DNA recognition as well.

P16^INK4A^ (CDKN2A/MTS1) is a cell cycle regulator involved in inhibition of the G1 phase progression [18]. Methylation of *p16* CpG islands silences transcription of this gene epigenetically in many cancers [19]. *p16* Methylation positively correlates with the risk of malignant transformation of human epithelial dysplasia [20,21]. In our recent work, a group of *p16-*promoter specific seven zinc-finger proteins (7ZFPs) were designed and fused with the activator VP64 domain to construct *p16*-specific artificial transcription factors (p16ATFs). These p16ATFs could re-activate expression of methylation-silenced *p16* through demethylation of CpG islands around the transcription start site of this gene [22]. Each of these 7ZFPs is composed of three-zinc-finger domain of the natural Sp1 (3ZF) in the *N*-terminal, two Sp1-like two-zinc-finger mutants derived from the Sp1 *finger-2* and −*3* in the middle and *C-*terminal regions. In the present study, a set of *N*-terminal, *C*-terminal, and linker variants for the representative 7ZFPs with *p16-*binding activity were further constructed, and used for comparison of contributions of different regions to the *p16*-binding activity.

## Methods

### Target p16 sequence, oligos, and probes

**Figure 1:**
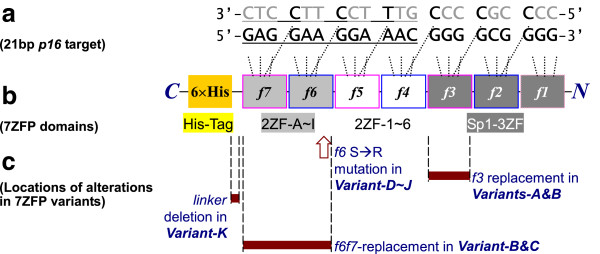
**Illustration of different 7ZFP variants. (a)** The 21 bp *p16* promoter target sequence, including the Sp1-binding site (9 bp) and its 5′-flanking sequence (12 bp, underlined); The zinc-finger directly binding deoxynucleotides are marked with bold characters; **(b)** Motifs in the artificial 7ZFP construction and their binding deoxynucleotides; fingers in pink or blue frames derived from Sp1 finger-3 and finger-2, respectively; **(c)** Locations of different alterations in the 7ZFP variants (deep red bars and arrow).

It has been reported that -622nt ~ −280nt region is the core promoter of *p16* gene and that the Sp1-binding site within -466nt ~ −451nt region (refer to the translation start site ATG) is crucial for transcription of this gene [23,24]. Thus, a 21 bp fragment within the core promoter (Figure 1a), including a natural Sp1-binding site (5′-ggggcgggg-3′) and its 5′-flank 12 bp sequence (5′-gaggaaggaaac-3′), was used as the target sequence to design *p16*-specific binding proteins of artificial 7ZFPs in our recent study [22]. The *p16* promoter-specific sequence of the biotin-labelled and unlabelled double-strand DNA Probe-1 used in electrophoresis mobility shift assay (EMSA) was 5′-aaggt tgtat cgcgg aggaa ggaaa cgggg cgggg gcgga tttct-3′ that contains the 21 bp 7ZFP binding site. A *p16* 5′UTR-specific sequence containing a postulated Sp1-binding site was used as the control probe (Probe-2: 5′-agggg ctggc tggtc accag agggt ggggc ggacc gagtg cgctc-3′). These double-strand probes were purified by polyacrylamide gel (PAGE).\

### Construction of 7ZFPs

The designed *p16*-specific 7ZFPs were constructed as described before [22]. Briefly, the two-zinc-finger modules (2ZF) derived from the Sp1 zinc-*finger-2* and −*3* were obtained by overlap*-*PCR using synthesized oligo deoxynucleotides containing the coding sequence of zinc-fingers, and then ligated together with the *N-*terminal 3ZF modules by particular restriction enzyme (Figure 1b). Furthermore, various *N*-terminal and *C*-terminal finger or linker variants were respectively constructed for the representative 7ZFPs that could bind with the *p16* promoter specifically using various oligos DNA (Figure 1c and Table 1). All of these 7ZFPs and their variants were inserted into T-cloning vector and confirmed by sequencing.

**Table 1:** List of the target triplets, amino acid residues at the position −1 ~ +6 of the α-helix of each single-zinc-finger domain of active 7ZFPs and their binding activity

**Active 7ZFPs and their variants marked with the line number**	**p16 DNA binding activity**^**a**^	***N*-terminal *Sp1 *** **three-zinc-finger domain (3ZF) and its mutants**			**Middle *Sp1-like *** **two-zinc-finger modules (2ZF)**		***C*-terminal *Sp1-like *** **two-zinc-finger modules (2ZF)**		***C*-terminal *linker *** **between *finger-7 *** **&6 × His-tag**
		***finger-1***	***finger-2***	***finger-3***	***finger-4***	***finger-5***	***finger-6***	***finger-7***	
		**5′-ggg-3′**	**5′-gcg-3′**	**5′-ggg-3′**	**5′-aac-3′**	**5′-gga-3′**	**5′-gaa-3′**	**5′-gag-3′**	
**● Finger motif replacement**									
1**_*Active-A *(consensus)**^b^	(++)	KTSHLRA	RSDELQR	RSDHLSK	ESDNLSQ	QSGHLQR	QSSNLQR	RSDNLAR	TGEKP
2_*Variant-A (f3)*	(++)	−−−−−−− ^c^	−−−−−−−	Q−G−−QR	−−−−−−−	−−−−−−−	−−−−−−−	−−−−−−−	−−−−−
3**_*Active-B***	(++)	−−−−−−−	−−−−−−−	−−−−−−−	−−−−−−−	−−−−−−−	−−−−−−−	−−−−−T−	−−−−−
4_*Variant-B (f3)*	(++)	−−−−−−−	−−−−−−−	Q−G−−QR	−−−−−−−	−−−−−−−	−−−−−−−	−−−−−T−	−−−−−
5_*Variant-B (f6f7)*	(−)	−−−−−−−	−−−−−−−	−−−−−−−	−−−−−−−	−−−−−−−	D−G−−R−	Q−GH−Q−	−−−−−
6**_*Active-C***	(++)	−−−−−−−	−−−−−−−	−−−−−−−	−−−−−−−	−−T−−−−	−−−−−−−	−−−−−T−	−−−−−
7_*Variant-C (f6f7)*	(−)	−−−−−−−	−−−−−−−	−−−−−−−	−−−−−−−	−−T−−−−	D−G−−R−	Q−GH−Q−	−−−−−
**● Single key amino acid mutation**									
8**_*Active-D***	(+)	−−−−−−−	−−−−−−−	−−−−−−−	−−−−−−−	−−−−−−−	−−−−−V−	−−−A−Q−	−−−−−
9_*Variant-D (f6)*	(−)	−−−−−−−	−−−−−−−	−−−−−−−	−−−−−−−	−−−−−−−	−−R−−V−	−−−A−Q−	−−−−−
10**_*Active-F***	(++)	−−−−−−−	−−−−−−−	−−−−−−−	−−−−−−−	−−T−−−−	−−−−−V−	−−−−−T−	−−−−−
11_*Variant-F (f6*)^d^	(−)	−−−−−−−	−−−−−−−	−−−−−−−	−−−−−−−	−−T−−−−	−−R−−V −	−−−−−T−	−−−−−
12**_*Active-G***	(++)	−−−−−−−	−−−−−−−	−−−−−−−	D−G−−RV	−−−−−−−	−−−−−V−	−−−−−T−	−−−−−
13_*Variant-G (f6)*	(−)	−−−−−−−	−−−−−−−	−−−−−−−	D−G−−RV	−−−−−−−	−−R−−V −	−−−−−T−	−−−−−
14**_*Active-H***^c^	(+)	−−−−−−−	−−−−−−−	−−−−−−−	D−G−−RV	−−−−−−−	−−−−−V−	−−−A−Q−	−−−−−
15_Variant-H (f6)	(−)	−−−−−−−	−−−−−−−	−−−−−−−	D−G−−RV	−−−−−−−	−−R−−V−	−−−A−Q−	−−−−−
16**_*Active-I***	(++)	−−−−−−−	−−−−−−−	−−−−−−−	D−G−−RR	−−−−−−−	−−−−−V−	−−−−−T−	−−−−−
17_*Variant-I (f6)*	(−)	−−−−−−−	−−−−−−−	−−−−−−−	D−G−−RR	−−−−−−−	−−R−−V −	−−−−−T−	−−−−−
18**_*Active-J***	(+)	−−−−−−−	−−−−−−−	−−−−−−−	D−G−−RR	−−T−−−−	−−−−−V−	−−−A−Q−	−−−−−
19_*Variant-J (f6)*	(−)	−−−−−−−	−−−−−−−	−−−−−−−	D−G−−RR	−−T−−−−	−−R−−V−	−−−A−Q−	−−−−−
20**_*Active-K***	(++)	−−−−−−−	−−−−−−−	−−−−−−−	D−G−−RR	−−T−−−−	−−−−−V−	−−−−−T−	−−−−−
21_*Variant-K (f6)*	(−)	−−−−−−−	−−−−−−−	−−−−−−−	D−G−−RR	−−T−−−−	−−R−−V −	−−−−−T−	−−−−−
**● *C*-terminal linker deletion**									
22**_*Active-K***	(++)	−−−−−−−	−−−−−−−	−−−−−−−	−−−−−−−	−−−−−−−	−−−−−V−	−−−−−T−	−−−−−
23_*Variant-K (linker)*	(−)	−−−−−−−	−−−−−−−	−−−−−−−	−−−−−−−	−−−−−−−	−−−−−V−	−−−−−T−	TG

^a^analyzed in EMSA assays.

^b^the exact sequence of amino acids of 7ZFP *Active-A*: *N*-MASRDPGKKKQHICHIQGCGKVYGKTSHLRAHLRWHTGERPFMCTWSYCGKRFTRSDELQRHKRTHTGEKKFACPECPKRFMRSDHLSKHIKTHQTGEKPFMCTWSYCGKRFTESDNLSQHKRTHTGEKKFACPECPKRFMQSGHLQRHIKTHTGEKPFMCTWSYCGKRFTQSSNLQRHKRTHTGEKKFACPECPKRFMRSDNLARHIKTHTGEKPFLEHHHHHH-*C*.

^c^the symbol “−” means the amino acid residue is the same as that in the consensus sequence of *Active-A* at the same position in the same column. ^d^used in optimization assays for soluble 7ZFP preparation.

### Preparation of soluble 7ZFPs

7ZFP-coding sequences in T-cloning vectors were digested by *NcoI* and *XhoI* restriction enzymes, and then inserted into six different kinds of expression vectors including pET-28a, pET-30a, pET-32a, pMal-p2X, pGEX-4 T-1, and pQE-Trisystem. These constructs were transformed into the corresponding expression bacteria and cultured in LB medium overnight. After the density of the culture was adjusted to 0.5 OD_600nm_ with the fresh medium, 7ZFPs expression was induced by addition of IPTG (Genview, USA) to the medium (final concentration, 0.1 mM) under different temperatures for different culture times. The sonicated bacteria proteins were separated by SDS-PAGE and visualized by Coomassie Blue staining. The amounts of soluble 7ZFPs from different expression vectors were compared with each other to select an optimal expression vector for preparation of the soluble ZFPs as described below.

After the IPTG-induction of expression of 7ZFPs in the above tested vectors, the bacteria were pelleted by centrifugation, and then frozen at −80°C if necessary. The pellets were re-suspended in 2 ml cold PBS/0.1% NP-40 buffer per 10 ml bacteria culture and lysed by ultrasonication followed by centrifugation (10000 rpm for 10 min). The soluble 7ZFPs were purified from the sonicated supernatants through incubation with Ni-NTA-Sepharose beads (GE Healthcare, German) for 1 hr, and eluted from the beads with 500 mmol/L of imidazole, according to the protocol of the instruction manual. The purified proteins were subjected to electrophoresis and Western blot to confirm their size and purity.

### Western blot

The proteins were electrophoresed through 12% SDS-PAGE and then transferred onto PVDF membranes. After blocking with 5% fat-free milk overnight at 4°C, the primary monoclonal antibody against 6 × Histidine-tag (Beijing PuLiLai Company, China) were applied at 1:2000 dilutions for 1 hr at room temperature. After washing, the membrane was incubated with specific horseradish peroxidase-conjugated secondary antibody for 1 hr. The signals were visualized using the Enhanced Chemoluminescence kit (PIERCE, USA).

### Electrophoresis mobility shift assay (EMSA)

Light Shift Chemiluminescent EMSA Kit (PIERCE, USA) was used. The EMSA assay was carried out according to the protocol of the Kit instruction as described previously [22]. Briefly, 5 nmoles of the biotin-labelled *p16*-promoter specific probes was incubated with 2 μl of the tested protein for 30 min on ice in 1 × binding buffer (25 mM HEPES, 100 mM KCl, 1 mM EDTA, 10 mM MgCl_2_, 0.1% NP40, 1% glycerol, 1 mM DTT). The reaction mixture were run on a 7% polyacrylamide/0.5 × TBE gel containing 2.5% glycerol at 100 V for 80 min and then transferred onto Hybond N + nylon membrane (Amersham, USA). In the competition assay, 25-, 200-, and 400-fold excesses of the corresponding unlabeled probes were pre-incubated with the tested proteins for 30 min prior to the addition of the labelled probes.

## Results

### Design and construction of 7ZFP variants

To evaluate the contribution of each domain in active artificial ZFPs to DNA binding activity, sets of 7ZFPs variants containing key amino acid mutations, finger replacements, and linker deletion in the *C*-terminal and *N*-terminal regions were designed and constructed respectively for the representative 7ZFPs which strongly bind to the *p16* target DNA (Figure 1), according to our recently report [22]. These variants includes, [I] two kinds of finger-replacement mutants in the *N*-terminal 3ZF and *C*-terminal 2ZF domains [Table 1, lines 2 ~ 7: *Variant-A/B(f3)* and *Variant-B/C(f6f7)*]; [II] two sets of mutants containing a S > R amino acid mutation at position +2 of *finger-6* in the *C*-terminal 2ZF domain (Table 1, lines 8 ~ 21, *Variant-D* ~ *J*); and [III] a linker mutant with a deletion of three amino acids (TGEKP> TG) between *finger-7* and 6×Histidine-tag in the *C*-terminal region (Table 1, lines 22 ~ 23, *Variant-K*).

### Optimisation of conditions for soluble 7ZFP preparation

**Figure 2:**
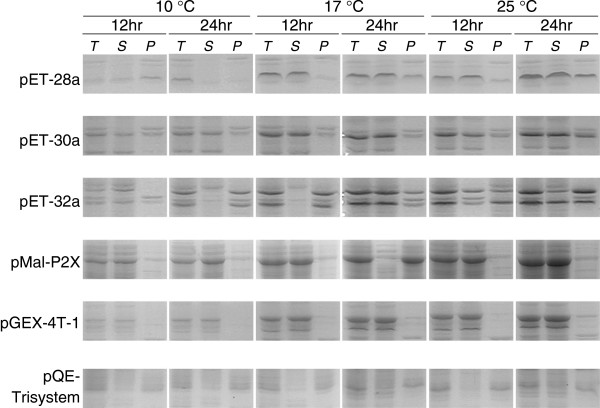
**Comparison of the expression states of the soluble 7ZFP *Active-H *** **in various kinds of vectors under different culture temperatures and IPTG-induction times (*T*: whole bacteria; *S*: the supernatant; *P*: precipitate).**

**Figure 3:**
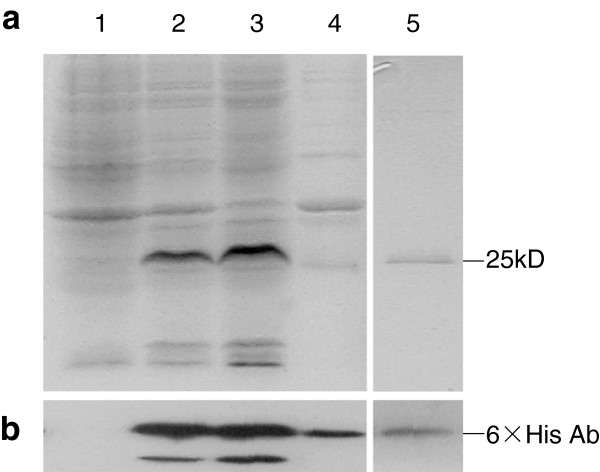
**Total protein and purified 7ZFP *Active-H *in different preparations from Rosetta DE3 bacteria transformed with the pET-28 7ZFP or empty control vectors. (a)** Total crude proteins expressing 7ZFP (line-pointed band), Coomassie Blue staining. **(b)** The expression of 7ZFP *Active-D* was displayed in Western blot assay using antibody against 6 × Histidine-tag (Lane-**1**, total protein for pET-28a control vector; Lane-**2**, total protein for pET-28a-7ZFP vector; Lane-**3**, the sonicated supernatant for pET-28a-7ZFP vector; Lane-**4**, the sonicated precipitate for pET-28a-7ZFP vector; Lane-**5**, 7ZFP purified from the sonicated supernatant using Ni-NTA-Sepharose beads).

To obtain soluble 7ZFPs, two distinct representative 7ZFPs [*Active-H* and *Variant-F(f6)* with the maximum number of amino acid differences] were inserted into six different kinds of expression vectors, including pET-28a, pET-30a, pET-32a, pMal-p2X, pGEX-4T-1, and pQE-Trisystem. Following transforming these vectors, the expression level and percentage of the soluble 7ZFPs in the corresponding bacteria were compared under various culture temperatures (10°C, 17°C, and 25°C) and IPTG induction times (12 hr and 24 hr). Results showed that soluble *Active-H* was most efficiently expressed in the pET-28a vector-transfected Rosetta (DE3) bacteria, after treatment of 0.1 mM of IPTG at 17°C for 24 hr (Figure 2). Most 7ZFPs expressed in the pET-28a vector were dissolved in the sonicated supernatants (about 25 kD). Similar results were obtained for *Variant-F(f6)* (data not shown). Therefore, the pET-28a vector was used to express the soluble 7ZFPs for further experiments. After purification using Ni-NTA-Sepharose beads, the soluble 7ZFPs were detected as a marked specific band and confirmed by Western blot (Figure 3). Such a band was not observed in the uninduced or pET-28a transformed-control bacteria.

### Comparison of DNA binding affinity and specificity of 7ZFPs with their variants

**Figure 4:**
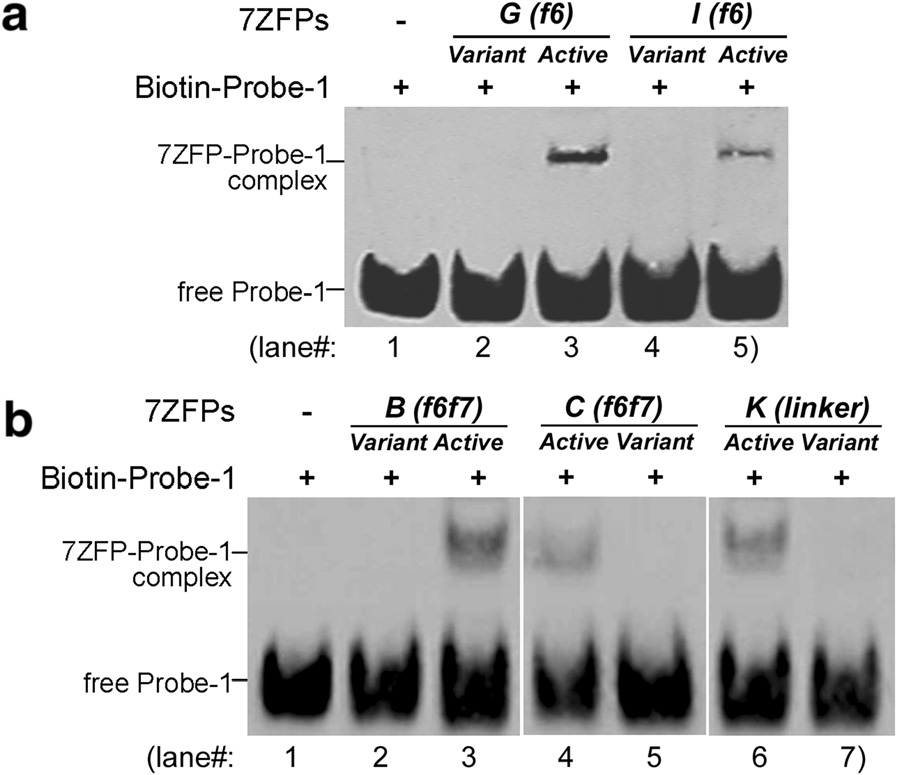
**Results of EMSA assay for the binding of the purified 7ZFPs to the *p16*-specific Probe-1. (a)** The binding activity of 7ZFPs to the Probe-1 DNA was abolished after the S to R mutation in *finger 6* in the 7ZFPs *Active-G* and *Active-I*; *Variant-(f6)*, variants with a S to R mutation of the key amino acid at the position +2 of the α-helix in *finger-6* in 7ZFPs; **(b)** The binding activity of 7ZFPs to the Probe-1 DNA was abolished when their *C*-terminal 2ZF domain in was replaced with the middle 2ZF domain in the 7ZFP *Active-B* and *Active-C*, or the linker between *finger-7* and the 6Histidine-tag in the *C*-terminal region was shorten from TGEKP to TG in the 7ZFP *Active-K*.

**Figure 5:**
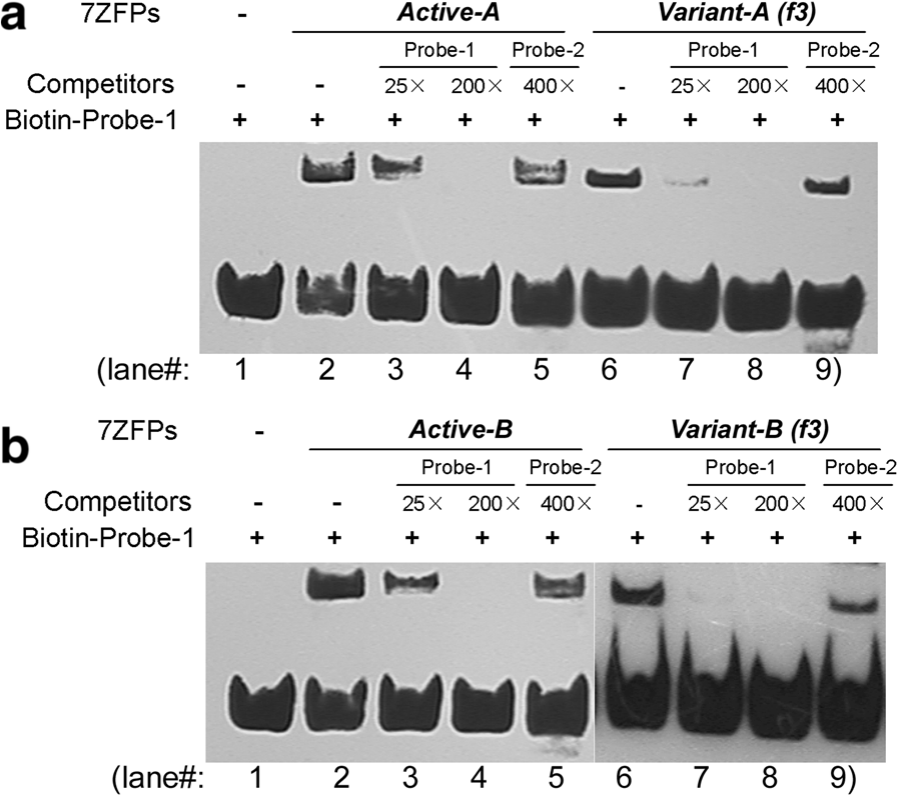
**Regular and competition EMSA assays for the binding of the purified 7ZFPs to the *p16 *promoter specific Probe-1. (a and b)** The binding activity of 7ZFPs to the Probe-1 DNA was partially abolished after *finger 3* (RSDHLSK) in the 7ZFPs *Active-A* and *Active-B* was replaced with *finger-5* (QSGHLQR). Probe-2 is the fragment of *p16* 5′UTR region containing a postulated Sp1 binding site.

As previously reported [22], results in the EMSA assays using the purified 7ZFPs showed that the representative 7ZFPs including *Active-A to -K* had high affinity binding to the *p16* promoter-specific Probe-1 (Figures 4, 5, and Table 1). First, we investigated whether substitution of single amino acid mutation (S > R) at position +2 of *finger-6*′s α-helix in the *C*-terminal 2ZF could affect the Probe-1 binding affinity. As shown in Figure 4a, compared with *Active-G* and *Active-I*, this substitution completely abolished their binding ability with Probe-1 (compare lane 2 and 3, lane 4 and 5, also see Table 1, line 12 to 13 and line 16 to 17). The EMSA experiments with 7ZFPs *Active-D*, *F*, *H*, *J*, *K* and their variants also showed the same results (EMSA image not shown, and Table 1, line 8 to 11, line 14 to 15, and line 18 to 21). Second, for 7ZFP *Active-K*, a deletion (TGEKP > TG) of 3 amino acid residues in the linker between *C*-terminal *finger-7* and the 6 × Histidine-tag also fully destroyed *p16* binding (Figure 4b, compare lane 6 and 7, Table 1, line 22 and 23). Next, full depletion of *p16* binding activity was also observed for the representative 7ZFPs *Active-B* and *Active-C* after their *finger-6* and −*7* were replaced with *finger-4* and −*5* (Figure 4b, compare lane 2 and 3, lane 4 and 5; Table 1, compare line 3 and 5, line 6 and 7). In contrast, the replacement of *finger-3* at the *N*-terminal 3ZF domain with *finger-5* in 7ZFPs *Active-A* and *Active-B* did not affect their *p16* binding affinity (Figure 5a, compare lane 2 and 6; Figure 5b, compare lane 2 and 6; Table 1, compare line 1 and 2, line 3 and 4). These results suggest that the *C-*terminal fingers and flanking sequence may play a crucial role in determining the 7ZFPs’ DNA binding affinity.

Competition EMSA assays using unlabeled Probe-1 and Probe-2 were further carried out to investigate whether the *N*-terminal domain in 7ZFPs contributes to the *p16-*binding specificity. Results showed that pre-incubation with the unlabeled *p16* promoter-specific Probe-1 (25×) completely blocked the binding of both 7ZFPs *Variant-A(f3)* and *Variant*-*B(f3)* to Probe-1 (Figure 5a, compare lane 6 and 7; Figure 5b, compare lane 6 and 7), but only partially decreased the binding of their active counterparts *Active-A* and *Active-B* to Probe-1 (Figure 5a, compare lane 2 and 3; Figure 5b, compare lane 2 and 3). Moreover, the pre-incubation with the unlabeled Probe-2 (a *p16* 5′UTR sequence containing a Sp1 binding site) did not decrease the DNA binding activity of 7ZFPs *Active-A*, *Active-B* (Figure 5a, lane 5 and Figure 5b, lane 5), nor their *N*-terminal 3ZF-variants *Variant-A(f3)* and *Variant*-*B(f3)* (Figure 5a, lane 9 and Figure 5b, lane 9). These results imply that 7ZFP *N*-terminal variants can specifically bind to the *p16* promoter target sequence with a decreased binding stability.

## Discussions

The engineered DNA-binding proteins can be utilized to carry out a variety of cellular activities by combining them with different functional domains. Researchers have successfully designed novel nucleases and transcription factors using the C2H2-type zinc finger domain as a scaffold [5-9]. However, the general principles of which zinc finger or which key amino acid in the engineered ZFPs play more important roles in the target binding are not well studied, especially for those containing multiple zinc fingers. In the present study, we found that the *C*-terminal region (finger and linker) in the artificial ZFPs played a more important role than the *N*-terminal region in determining their DNA binding affinity, and that the *N*-terminal fingers could stabilize their binding to the target DNA. Similar phenomena have previously been reported for several natural ZFPs [12-14]. However, to the best of our knowledge, this is the first report to show the functional difference between the *N*-terminal and *C*-terminal regions within the engineered ZFPs. These findings may be useful for ZFP engineering.

Sp1 is an important transcription factor that binds to several thousands of GC-boxes with the consensus sequence 5′-GGGGCGGGG-3′ in the genome. It is an ideal scaffold for adding more zinc-fingers to its three-zinc-finger domain and obtaining better gene-specificity for the engineered transcription factors because of its accessibility to GC-boxes within chromatin and low antigenicity. It has been reported that contribution of the *C*-terminal *finger-3* in natural C2H2 ZFPs to their DNA-binding affinity is higher than the *N*-terminal *fingers-1*, and further that *finger-1* can stabilize their DNA-binding [12-14]. In our study, *C*-terminal *finger 3*-in the natural Sp1 was used to construct 7ZFPs at its *N*-terminal. Interestingly, after the 7ZFP *finger-3* [RSDHLSK] in 7ZFPs *Active-A* and *Active-B* was replaced with the *finger-5* [QSGHLQR], these 7ZFP variants showed the same DNA binding affinity; however, their DNA binding stability was decreased. These results indicate that the functions of *finger-3* are changed after its location shifts from *C*-terminal in Sp1 to *N*-terminal in 7ZFPs. As in the natural Sp1, the *N*-terminal fingers in the Sp1-like artificial ZFPs may play additional roles in stabilization of their DNA binding and few roles in determining their DNA binding affinity.

Studies on Zif268 and artificial ZFPs have suggested that the amino acid at position +2 of the α-helix of each finger might be able to interact with the 4^th^ DNA base on the opposite strand of the flanking triplet DNA target, leading to synergy between adjacent fingers and enhancing specificity of the amino acids at other positions [15-17,25]. Amino acids with shorter and smaller side chains in artificial ZFPs are generally used for recognition of the 2^nd^ and 4^th^ DNA bases in artificial ZPF design [26]. Aspartic acid [D] and serine [S] exist frequently at position +2 of each α-helix in zinc-fingers. In order to investigate whether the *C*-terminal fingers in the Sp1-like artificial ZFPs remain important for the DNA-protein binding as observed in the natural Sp1, *C*-terminal 2ZF variants containing the key amino acid mutation [from serine (with a polar but un-charged short side chain) to arginine (with a charged long side chain), S > R] at position +2 of the α-helix in the *finger-6*, or whole 2ZF domain replacement (from the *C*-terminal 2ZF to the middle 2ZF) were constructed and used for evaluation of DNA binding affinity changes in EMSA assay. As expected, both the S > R mutation and *C*-terminal 2ZF replacement completely impaired the affinity of their corresponding 7ZFPs. These results show that the *C*-terminal domain in the Sp1-like artificial ZFPs remains important in determining the DNA binding affinity, as observed in the natural Sp1.

It has been reported that Kaiso uses all three fingers as well as the flanking sequences for high-affinity binding to target DNA and that extended linker may be helpful for the DNA binding of ZFPs [14,27]. In the present study, it was found that deletion of three amino acid residues in the linker between the *C*-terminal *finger-7* and 6 × Histidine-tag also completely abolished the binding affinity of 7ZFPs to *p16* promoter-specific DNA. This suggests that the proper flexibility of the adjacent amino acid sequence flanking the *C*-terminal fingers is also a determinant for the high DNA binding affinity of the Sp1-like ZFPs.

## Conclusions

The present study show for the first time that the *C-*terminal domain in the Sp1-like artificial ZFPs may play a crucial role in determining ZFPs’ binding affinity to the target DNA and that the *N-*terminal domain may help stabilizing this binding affinity.

## Competing interests

The authors declare that they have no competing interests.

## Authors’ contributions

BZ constructed the ZFP variants and performed the competition EMSA assays with the purified ZFPs and drafted the manuscript. SX designed the zinc-finger units and constructed the 7ZFPs vectors. YY performed the regular EMSA experiments. LG participated in the preparation and purification of 7ZFPs. DD conceived of the study and revised the manuscript. All authors read and approved the final manuscript.
